# Supplementary material for: The relative contributions of subjective and musical factors in music for sleep
Source: PLoS One. 2025 Aug 21;20(8):e0330268. doi: 10.1371/journal.pone.0330268 (PMC12370070; doi:10.1371/journal.pone.0330268)
Supplement: S1 Table — (DOCX) [file pone.0330268.s001.docx]

**S1. Music selection.**

1. **Spotify**

To generate the selection of music, twelve audio features provided by the Spotify API were extracted and a principal component analysis (PCA) reduced the variables to two components. Component scores were then used to characterise each track. After calculating scores, the dataset was re-divided into the different categories. Modified z-scores using Median Absolute Deviation (1) against the median within each category were calculated on each component score to re-centre these values within their respective groups. Using the explained variance percentages from the PCA for each component, weighted averages were calculated to give a single score for each track. The eight tracks nearest to 0 (positive and negative, four either side) in each group were selected on the assumption that they should be generally representative of each category. Three tracks in the Sleep category were only available for streaming on Spotify and thus could not be purchased for download, and one track had to be excluded at a later stage due to copyright restrictions when implementing in the online study. All tracks were purchased for download from iTunes or Amazon. Tracks in bold were omitted either due to being unavailable for purchase or were blocked from YouTube on the grounds of copyright issues; tracks in italics are the replacements. Further results in the Sleep playlist not being available outside of Spotify streaming resulted in a slight skew towards the negative side of 0 in this selection, but this was kept in the interest of remaining closer to 0 rather than simply having an equal number of results either side of 0.

| **Category** | **Artist** | **Album** | **Track** |
| --- | --- | --- | --- |
| **Energising** | *Clean Bandit* | *Tick Tock (feat. 24kGoldn)* | *Tick Tock (feat. 24kGoldn)* |
|  | **Bad Bunny** | **LAS QUE NO IBAN A SALIR** | **PA' ROMPERLA** |
|  | Spillage Village | Baptize (with JID & EARTHGANG feat. Ant Clemons) | Baptize (with JID & EARTHGANG feat. Ant Clemons) |
|  | 220 KID | Too Many Nights | Too Many Nights |
|  | Shawn Mendes | Shawn Mendes | In My Blood |
|  | The Weeknd | After Hours | Blinding Lights |
|  | Janet Jackson | Rhythm Nation 1814 | Rhythm Nation |
|  | Avicii | Stories | Waiting For Love |
|  | THAT KIND | Lights Go Down | Lights Go Down |
| **Relaxing** | Band of Horses | Acoustic at The Ryman (Live) | The Funeral - Live Acoustic |
|  | Nothingtosay | Introspective | For You |
|  | Healing Sounds for Deep Sleep and Relaxation | Spiritual Shamanic Music – 15 Ambient Songs Perfect for Deep Meditation and Sleep | Ethnic Session |
|  | xander. | Cabin Fever | Don't Let Her Go |
|  | No Spirit | Memories We Made | Some Alone Time |
|  | Ryohei Shimoyama | Winter Milky Way | Winter Milky Way |
|  | Sitting Duck | Wonderland Chapter 1 | Slow Mornings |
|  | S N U G | Moonglow | Missing You |
| **Sleep** | *Alice ASMR* | *ASMR Trigger Sounds* | *Blow Torch ASMR* |
|  | *Dan Evans-Parker* | *Hush* | *Hush* |
|  | Max Huber | When You Love Someone (Piano Version) | When You Love Someone - Piano Version |
|  | Bud Hollister | The Stillness Within | The Stillness Within |
|  | Pacific Ocean Samples | Beach Waves | White Noise Waves |
|  | **Luana Dias Araujo** | **Polly Wolly Doodle** | **Polly Wolly Doodle** |
|  | Steve Devon | Only Trust Your Heart | Only Trust Your Heart |
|  | ThePianoPlayer | Sonnambula | Sogni d'oro |
|  | **Carla Moses** | **I Will Say Goodbye** | **smoke gets in your eyes** |
|  | **Serge Charlesbois** | **Hot Cross Buns** | **Rub-A-Dub Dub Three Men In A Tub** |
|  | *Ron Adelaar* | *Gymnopédie No.1* | *Gymnopédie No.1* |

1. **Commercial sleep music**

| **Artist** | **Album** | **Track** | **Used in study** |
| --- | --- | --- | --- |
| Dr. Jeffrey Thompson | Delta Sleep System | Delta Sleep System, Part 1 | (2) |
| Dr. Lee Bartel / SonicAid | Music to Promote Sleep | Drifting into Delta | (3,4) |
| Marconi Union | The Ambient Zone Just Music Café, Vol. 4 | Weightless | (5,6) |
| Max Richter | From Sleep | Dream 3 (in the midst of my life) | (7) – used the longer ‘Sleep’ album. |
|  |  | Dream 13 (minus Even) |  |
| Niels Eje | MusiCure | The North | (8) |
|  |  | Legend |  |
|  |  | Northern Light |  |

**Supplementary References**

1. Leys C, Ley C, Klein O, Bernard P, Licata L. Detecting outliers: do not use standard deviation around the mean, use absolute deviation around the median. Journal of Experimental Social Psychology. 2013 Jul 1;49(4):764–6.

2. Lazic SE, Ogilvie RD. Lack of efficacy of music to improve sleep: a polysomnographic and quantitative EEG analysis. International Journal of Psychophysiology. 2007 Mar 1;63(3):232–9.

3. Cordi MJ, Ackermann S, Rasch B. Effects of relaxing music on healthy sleep. Scientific Reports. 2019 Jun 24;9(1):1–9.

4. Picard LM, Bartel LR, Gordon AS, Cepo D, Wu Q, Pink LR. Music as a sleep aid in fibromyalgia. Pain Research and Management. 2014;19(2):97–101.

5. Shepherd D, Hautus MJ, Giang E, Landon J. “The most relaxing song in the world”? A comparative study. Psychology of Music. 2023 Jan 1;51(1):3–15.

6. Graff V, Cai L, Badiola I, Elkassabany NM. Music versus midazolam during preoperative nerve block placements: a prospective randomized controlled study. Reg Anesth Pain Med. 2019 Aug 1;44(8):796–9.

7. Kuula L, Halonen R, Kajanto K, Lipsanen J, Makkonen T, Peltonen M, et al. The effects of presleep slow breathing and music listening on polysomnographic sleep measures – a pilot trial. Scientific Reports. 2020 May 4;10(1):7427.

8. Jespersen KV, Vuust P. The effect of relaxation music listening on sleep quality in traumatized refugees: a pilot study. J Music Ther. 2012 Jul 1;49(2):205–29.
