# Supplementary material for: The relative contributions of subjective and musical factors in music for sleep
Source: PLoS One. 2025 Aug 21;20(8):e0330268. doi: 10.1371/journal.pone.0330268 (PMC12370070; doi:10.1371/journal.pone.0330268)
Supplement: S2 Table — (DOCX) [file pone.0330268.s002.docx]

**S2. Additional participant information**

| **Scale/measure** | **Items** | **Mean** | **Median** | **SD** | **Minimum** | **Maximum** |
| --- | --- | --- | --- | --- | --- | --- |
| **Short Big-Five** | **Extroversion** | 4.185 | 4.50 | 1.789 | 1 | 7 |
|  | **Agreeableness** | 5.667 | 6.00 | 1.275 | 1 | 7 |
|  | **Conscientiousness** | 5.389 | 6.00 | 1.317 | 1 | 7 |
|  | **Emotional stability** | 4.917 | 5.00 | 1.505 | 1 | 7 |
|  | **Openness to experience** | 5.824 | 6.00 | 1.126 | 3 | 7 |
| **STOMP factors** | **Mellow** | 4.731 | 5.00 | 1.791 | 1 | 7 |
|  | **Unpretentious** | 4.241 | 4.33 | 1.263 | 1.000 | 7.00 |
|  | **Sophisticated** | 4.900 | 5.00 | 1.218 | 2.000 | 7.00 |
|  | **Intense** | 4.525 | 4.67 | 1.317 | 1.000 | 6.67 |
|  | **Contemporary** | 4.486 | 4.50 | 1.459 | 1.000 | 7.00 |
| **PSQI scores** | **Subjective sleep quality** | 1.222 | 1.00 | 0.688 | 0 | 3 |
|  | **Latency** | 1.528 | 1.00 | 1.018 | 0 | 3 |
|  | **Sleep duration** | 0.537 | 0.00 | 0.790 | 0 | 3 |
|  | **Habitual sleep efficiency** | 0.626 | 0 | 0.819 | 0 | 3 |
|  | **Sleep disturbance** | 1.176 | 1.00 | 0.470 | 0 | 2 |
|  | **Use of medications** | 0.315 | 0.00 | 0.805 | 0 | 3 |
|  | **Daytime disfunction** | 1.241 | 1.00 | 0.735 | 0 | 3 |
|  | **Global PSQI Score** | 6.639 | 6.00 | 3.254 | 0 | 17 |
| **Self-help methods for sleep** | **Reading** | 2.80 | 3.00 | 1.166 | 1 | 5 |
|  | **Podcasts** | 1.79 | 1.00 | 1.111 | 1 | 5 |
|  | **Music** | 2.34 | 2.00 | 1.209 | 1 | 5 |
|  | **TV** | 1.98 | 2.00 | 1.168 | 1 | 5 |
|  | **Meditation** | 1.98 | 1.00 | 1.207 | 1 | 5 |
|  | **Qigong** | 1.06 | 1.00 | 0.438 | 1 | 5 |
|  | **Breathing exercises** | 2.21 | 2.00 | 1.223 | 1 | 5 |
|  | **White/pink noise** | 1.68 | 1.00 | 1.167 | 1 | 5 |
|  | **Prayer** | 1.50 | 1.00 | 1.028 | 1 | 5 |
|  | **Snacks food** | 1.44 | 1.00 | 0.835 | 1 | 5 |
|  | **Natural sounds** | 2.19 | 2.00 | 1.320 | 1 | 5 |
|  | **Prescribed medication** | 1.31 | 1.00 | 0.779 | 1 | 5 |
|  | **Relaxation therapy** | 1.40 | 1.00 | 0.796 | 1 | 4 |
|  | **Light exercise** | 1.52 | 1.00 | 0.932 | 1 | 5 |
|  | **Aroma** | 1.56 | 1.00 | 0.989 | 1 | 5 |
|  | **Warmwater footbath** | 1.26 | 1.00 | 0.728 | 1 | 5 |
|  | **Tai chi** | 1.14 | 1.00 | 0.571 | 1 | 5 |
|  | **Natural products** | 1.64 | 1.00 | 1.063 | 1 | 5 |
|  | **Maintain lifestyle regularity** | 2.98 | 3.00 | 1.223 | 1 | 5 |
|  | **Massage** | 1.42 | 1.00 | 0.908 | 1 | 5 |
|  | **Mindfullness** | 1.96 | 1.50 | 1.215 | 1 | 5 |
|  | **OTC products** | 1.40 | 1.00 | 0.864 | 1 | 5 |
|  | **Alcohol** | 1.44 | 1.00 | 0.835 | 1 | 4 |
|  | **Yoga** | 1.49 | 1.00 | 0.942 | 1 | 5 |
|  | **Acupuncture** | 1.05 | 1.00 | 0.252 | 1 | 3 |
|  | **Herbs** | 1.50 | 1.00 | 1.028 | 1 | 5 |
|  | **Cupping** | 1.06 | 1.00 | 0.369 | 1 | 4 |
|  | **Bath** | 1.99 | 2.00 | 1.072 | 1 | 5 |
|  | **Audiobooks** | 1.65 | 1.00 | 1.053 | 1 | 5 |
|  | **Radio** | 1.50 | 1.00 | 0.912 | 1 | 4 |
|  | **Silence** | 3.44 | 4.00 | 1.423 | 1 | 5 |
|  | **Other** | 1.71 | 1.00 | 1.361 | 1 | 5 |
| **Use of passive methods** | **Audiobooks** | 1.49 | 1.00 | 0.791 | 1 | 4 |
|  | **Podcasts** | 1.65 | 1.00 | 0.857 | 1 | 4 |
|  | **Radio** | 1.39 | 1.00 | 0.759 | 1 | 4 |
|  | **TV** | 1.73 | 2.00 | 0.718 | 1 | 3 |
|  | **Natural sounds** | 1.95 | 1.00 | 1.131 | 1 | 4 |
|  | **White/pink noise** | 1.67 | 1.00 | 1.094 | 1 | 4 |
|  | **Music** | 1.88 | 2.00 | 0.817 | 1 | 4 |
|  | **Silence** | 3.29 | 4.00 | 1.144 | 1 | 4 |
|  | **Other** | 1.37 | 1.00 | 0.816 | 1 | 4 |
| **Reasons for using** | **Physically relax** | 1.98 | 2.00 | 0.843 | 1 | 5 |
|  | **Mentally relax** | 1.79 | 2.00 | 0.821 | 1 | 5 |
|  | **Distracts from stress** | 2.03 | 2.00 | 1.000 | 1 | 5 |
|  | **Reduce worry about next day** | 2.66 | 3.00 | 1.153 | 1 | 5 |
|  | **Improves mood** | 2.10 | 2.00 | 0.906 | 1 | 5 |
|  | **Blocks other sound** | 2.74 | 3.00 | 1.314 | 1 | 5 |
|  | **Think about day ahead** | 3.69 | 4.00 | 1.011 | 1 | 5 |
|  | **Reflect on day** | 3.26 | 3.00 | 1.062 | 1 | 5 |
|  | **Triggers memories** | 3.73 | 4.00 | 1.107 | 1 | 5 |
|  | **Enter alternative state** | 2.90 | 3.00 | 1.222 | 1 | 5 |
| **Mood** | **Valence** | 6.32 | 7.00 | 2.02 | 1 | 9 |
|  | **Tension** | 6.07 | 6.00 | 2.04 | 1 | 9 |
|  | **Alertness** | 5.20 | 6.00 | 1.97 | 1 | 9 |
