# Supplementary material for: The relative contributions of subjective and musical factors in music for sleep
Source: PLoS One. 2025 Aug 21;20(8):e0330268. doi: 10.1371/journal.pone.0330268 (PMC12370070; doi:10.1371/journal.pone.0330268)
Supplement: S3 Table — (DOCX) [file pone.0330268.s003.docx]

**S3. Further statistical results**

1. **Ratings comparisons**
   1. Sphericity tests for one-way repeated measures ANOVAs.

| **Rating** | **Mauchly's W** | **Approx. Chi-Square** | **df** | **Sig.** | **Epsilon** | | |
| --- | --- | --- | --- | --- | --- | --- | --- |
|  |  |  |  |  | **Greenhouse-Geisser** | **Huynh-Feldt** | **Lower-bound** |
| **Positive** | .946 | 5.855 | 2 | .054 | .949 | .966 | .500 |
| **Tense** | .931 | 7.600 | 2 | .022 | .935 | .951 | .500 |
| **Awake** | .960 | 4.282 | 2 | .118 | .962 | .979 | .500 |
| **Familiar** | .999 | .118 | 2 | .943 | .999 | 1.000 | .500 |
| **Engaging** | .868 | 15.000 | 2 | .001 | .883 | .897 | .500 |
| **Pleasant** | .963 | 4.024 | 2 | .134 | .964 | .981 | .500 |
| **Activating** | .952 | 5.240 | 2 | .073 | .954 | .971 | .500 |
| **Energising** | .917 | 9.216 | 2 | .010 | .923 | .939 | .500 |
| **Comforting** | .979 | 2.285 | 2 | .319 | .979 | .997 | .500 |
| **Absorbing** | .956 | 4.818 | 2 | .090 | .957 | .974 | .500 |
| **Distracting** | .886 | 12.828 | 2 | .002 | .898 | .912 | .500 |
| **Sleep Preventing** | .934 | 7.193 | 2 | .027 | .938 | .955 | .500 |
| **Liked** | .961 | 4.233 | 2 | .120 | .962 | .980 | .500 |

- 1. Repeated measures ANOVA results. Greenhouse-Geisser corrected results are presented in cases of sphericity violation.

| **Rating** | **Type III Sum of Squares** | **df** | **Error df** | **Mean Square** | **F** | **Sig.** | **Partial Eta Squared** |
| --- | --- | --- | --- | --- | --- | --- | --- |
| **Positive** | .668 | 2 | 214 | .334 | .300 | .741 | .003 |
| **Tense** | 289.901 | 1.871 | 200.152 | 154.979 | 118.246 | .000 | .525 |
| **Awake** | 615.401 | 2 | 214 | 307.700 | 232.227 | .000 | .685 |
| **Familiar** | 5.567 | 2 | 214 | 2.783 | 2.395 | .094 | .022 |
| **Engaging** | 16.973 | 1.767 | 189.054 | 9.606 | 5.922 | .005 | .052 |
| **Pleasant** | 23.277 | 2 | 214 | 11.639 | 9.335 | .000 | .080 |
| **Activating** | 631.704 | 2 | 214 | 315.852 | 244.675 | .000 | .696 |
| **Energising** | 505.965 | 1.846 | 197.550 | 274.049 | 222.335 | .000 | .675 |
| **Comforting** | 78.456 | 2 | 214 | 39.228 | 40.246 | .000 | .273 |
| **Absorbing** | 31.818 | 2 | 214 | 15.909 | 15.074 | .000 | .123 |
| **Distracting** | 211.038 | 1.795 | 192.104 | 117.546 | 69.874 | .000 | .395 |
| **Sleep Preventing** | 596.245 | 1.877 | 200.824 | 317.682 | 234.535 | .000 | .687 |
| **Liked** | 27.235 | 2 | 214 | 13.617 | 8.996 | .000 | .078 |

- 1. Pairwise post hoc tests.

| **Rating** | **Category A** | **Category B** | **MD (A-B)** | **Std. Error** | **Sig.** | **95% CI** | |
| --- | --- | --- | --- | --- | --- | --- | --- |
|  |  |  |  |  |  | **LB** | **UB** |
| **Positive** | Energising | Relaxing | -.015 | .143 | 1.000 | -.364 | .333 |
|  |  | Sleep | -.103 | .158 | 1.000 | -.486 | .280 |
|  | Relaxing | Sleep | -.088 | .129 | 1.000 | -.400 | .225 |
| **Tense** | Energising | Relaxing | 1.877 | .151 | <.001 | 1.510 | 2.243 |
|  |  | Sleep | 2.115 | .167 | <.001 | 1.709 | 2.521 |
|  | Relaxing | Sleep | .239 | .132 | .223 | -.083 | .561 |
| **Awake** | Energising | Relaxing | 2.639 | .162 | <.001 | 2.244 | 3.034 |
|  |  | Sleep | 3.143 | .166 | <.001 | 2.739 | 3.546 |
|  | Relaxing | Sleep | .504 | .140 | .002 | .163 | .845 |
| **Familiar** | Energising | Relaxing | .281 | .144 | .162 | -.070 | .632 |
|  |  | Sleep | .006 | .148 | 1.000 | -.354 | .365 |
|  | Relaxing | Sleep | -.275 | .148 | .198 | -.635 | .085 |
| **Engaging** | Energising | Relaxing | .498 | .159 | .007 | .111 | .884 |
|  |  | Sleep | .472 | .188 | .041 | .014 | .930 |
|  | Relaxing | Sleep | -.025 | .137 | 1.000 | -.360 | .309 |
| **Pleasant** | Energising | Relaxing | -.421 | .151 | .019 | -.787 | -.054 |
|  |  | Sleep | -.647 | .165 | <.001 | -1.047 | -.246 |
|  | Relaxing | Sleep | -.226 | .139 | .322 | -.565 | .113 |
| **Activating** | Energising | Relaxing | 2.521 | .168 | <.001 | 2.112 | 2.930 |
|  |  | Sleep | 3.262 | .156 | <.001 | 2.883 | 3.641 |
|  | Relaxing | Sleep | .742 | .139 | <.001 | .405 | 1.078 |
| **Energising** | Energising | Relaxing | 2.274 | .153 | <.001 | 1.902 | 2.646 |
|  |  | Sleep | 2.912 | .157 | <.001 | 2.529 | 3.294 |
|  | Relaxing | Sleep | .638 | .123 | <.001 | .339 | .936 |
| **Comforting** | Energising | Relaxing | -.837 | .132 | <.001 | -1.159 | -.515 |
|  |  | Sleep | -1.170 | .143 | <.001 | -1.518 | -.821 |
|  | Relaxing | Sleep | -.332 | .127 | .030 | -.641 | -.024 |
| **Absorbing** | Energising | Relaxing | -.577 | .139 | <.001 | -.915 | -.239 |
|  |  | Sleep | -.727 | .152 | <.001 | -1.098 | -.356 |
|  | Relaxing | Sleep | -.150 | .127 | .722 | -.458 | .159 |
| **Distracting** | Energising | Relaxing | 1.511 | .156 | <.001 | 1.132 | 1.889 |
|  |  | Sleep | 1.860 | .193 | <.001 | 1.390 | 2.330 |
|  | Relaxing | Sleep | .349 | .149 | .065 | -.015 | .712 |
| **Sleep Preventing** | Energising | Relaxing | 2.350 | .164 | <.001 | 1.951 | 2.750 |
|  |  | Sleep | 3.209 | .162 | <.001 | 2.816 | 3.603 |
|  | Relaxing | Sleep | .859 | .132 | <.001 | .537 | 1.181 |
| **Liked** | Energising | Relaxing | -.429 | .163 | .029 | -.825 | -.033 |
|  |  | Sleep | -.705 | .183 | .001 | -1.149 | -.260 |
|  | Relaxing | Sleep | -.276 | .155 | .237 | -.653 | .102 |
| Bonferroni adjustment for multiple comparisons. | | | | | | | |

1. **Features comparisons**
   1. Results of independent samples Kruskal-Wallis tests.

| **Feature** |  | **K-W H** | **df** | **Sig.** |
| --- | --- | --- | --- | --- |
|  | **N** |  |  |  |
| **Tempo** | 54 | .804 | 2 | .669 |
| **Pulse Clarity** | 54 | 20.871 | 2 | <.001 |
| **Event Density** | 54 | 21.818 | 2 | <.001 |
| **Mode** | 54 | .518 | 2 | .772 |
| **Key Clarity** | 54 | 8.166 | 2 | .017 |
| **Brightness** | 54 | 22.708 | 2 | <.001 |
| **Dynamic Energy** | 54 | 9.709 | 2 | .008 |
| **Dynamic Variation** | 54 | 6.143 | 2 | .046 |
| **Articulation** | 54 | 7.583 | 2 | .023 |

- 1. Pairwise post hoc tests for significant results.

| **Rating** | **Category A** | **Category B** | **Test Statistic** | **Std. Error** | **Std. Test Statistic** | **Sig.** |
| --- | --- | --- | --- | --- | --- | --- |
| **Pulse Clarity** | Energising | Relaxing | 19.900 | 5.654 | 3.520 | .001 |
|  |  | Sleep | 22.239 | 5.121 | 4.342 | <.001 |
|  | Relaxing | Sleep | 2.339 | 5.221 | .448 | 1.000 |
| **Event Density** | Energising | Relaxing | 20.875 | 5.654 | 3.693 | .001 |
|  |  | Sleep | 22.484 | 5.121 | 4.391 | <.001 |
|  | Relaxing | Sleep | 1.609 | 5.221 | .308 | 1.000 |
| **Key Clarity** | Energising | Relaxing | -9.313 | 5.654 | -1.647 | .299 |
|  |  | Sleep | -14.617 | 5.121 | -2.854 | .013 |
|  | Relaxing | Sleep | -5.304 | 5.221 | -1.016 | .929 |
| **Brightness** | Energising | Relaxing | 12.838 | 5.654 | 2.270 | .070 |
|  |  | Sleep | 24.351 | 5.121 | 4.755 | <.001 |
|  | Relaxing | Sleep | 11.513 | 5.221 | 2.205 | .082 |
| **Dynamic Energy** | Energising | Relaxing | 7.642 | 5.654 | 1.352 | .530 |
|  |  | Sleep | 15.853 | 5.121 | 3.095 | .006 |
|  | Relaxing | Sleep | 8.212 | 5.221 | 1.573 | .347 |
| **Dynamic Variation** | Energising | Relaxing | 2.454 | 5.654 | .434 | 1.000 |
|  |  | Sleep | 11.753 | 5.121 | 2.295 | .065 |
|  | Relaxing | Sleep | 9.299 | 5.221 | 1.781 | .225 |
| **Articulation** | Energising | Relaxing | -9.654 | 5.654 | -1.707 | .263 |
|  |  | Sleep | 4.682 | 5.121 | .914 | 1.000 |
|  | Relaxing | Sleep | 14.336 | 5.221 | 2.746 | .018 |
| Bonferroni adjustment for multiple comparisons. | | | | | | |

1. **Interrelations between subjective ratings**

Correlation matrix.

|  | **Positive** | **Tense** | **Awake** | **Familiar** | **Engaging** | **Pleasant** | **Activating** | **Energising** | **Comforting** | **Absorbing** | **Distracting** | **Sleep Preventing** |
| --- | --- | --- | --- | --- | --- | --- | --- | --- | --- | --- | --- | --- |
| **Tense** | -.570*** | — |  |  |  |  |  |  |  |  |  |  |
| **Awake** | -.174*** | .621*** | — |  |  |  |  |  |  |  |  |  |
| **Familiar** | .349*** | -.264*** | -.141*** | — |  |  |  |  |  |  |  |  |
| **Engaging** | .472*** | -.216*** | .084** | .307*** | — |  |  |  |  |  |  |  |
| **Pleasant** | .683*** | -.630*** | -.320*** | .443*** | .588*** | — |  |  |  |  |  |  |
| **Activating** | -.358*** | .736*** | .743*** | -.166*** | -.072** | -.515*** | — |  |  |  |  |  |
| **Energising** | -.110*** | .542*** | .759*** | -.066* | .152*** | -.221*** | .738*** | — |  |  |  |  |
| **Comforting** | .651*** | -.743*** | -.462*** | .381*** | .420*** | .814*** | -.653*** | -.377*** | — |  |  |  |
| **Absorbing** | .568*** | -.550*** | -.293*** | .385*** | .549*** | .792*** | -.462*** | -.219*** | .776*** | — |  |  |
| **Distracting** | -.413*** | .629*** | .552*** | -.255*** | -.228*** | -.565*** | .628*** | .531*** | -.660*** | -.550*** | — |  |
| **Sleep Preventing** | -.327*** | .689*** | .766*** | -.157*** | -.078** | -.489*** | .818*** | .725*** | -.646*** | -.470*** | .659*** | — |
| **Liked** | .612*** | -.568*** | -.280*** | .426*** | .650*** | .854*** | -.451*** | -.183*** | .780*** | .799*** | -.576*** | -.473*** |
| * p < .05, ** p < .01, *** p < .001 | | | | | | | | | | | | |
