# Supplementary material for: The relative contributions of subjective and musical factors in music for sleep
Source: PLoS One. 2025 Aug 21;20(8):e0330268. doi: 10.1371/journal.pone.0330268 (PMC12370070; doi:10.1371/journal.pone.0330268)
Supplement: S4 Table — (DOCX) [file pone.0330268.s004.docx]

**S4. List and description of ten most sleep-inducing pieces**

| **Piece** | **Source** | **Category** | **Description** | **Tempo** | **Tonality** |
| --- | --- | --- | --- | --- | --- |
| 1. SC-8 | Composers | Sleep | Solo piano, minimal classical, open ringing notes and chords | Slow/ free time | Maj |
| 1. Max Richter – Dream 13 (minus even) | CSM | Sleep | Piano and cello, classical | Med | Maj |
| 1. Niels Eje – The North | CSM | Sleep | Wave sounds, harp and piano, New Age | Med | Maj |
| 1. SC-13 | Composers | Relaxing | Solo piano, continuous melody | Slow | Min |
| 1. Erik Satie – Gymnopedie no.1 (Ron Adelaar) | Spotify | Sleep | Solo piano, classical | Slow | Maj |
| 1. Steve Devon – Only Trust Your Heart | Spotify | Sleep | Solo piano, slow jazz, continuous melody | Slow | Maj/7ths/dom |
| 1. Dan Evans-Parker – Hush | Spotify | Sleep | Piano and brushed snare, chord based | Slow | Min |
| 1. SC-14 | Composers | Relaxing | Cello, piano, clarinet, slow melody | Slow | Maj |
| 1. Max Richter – Dream 3 (in the midst of my life) | CSM | Sleep | Solo piano, chord based | Slow | Min |
| 1. Ryohei Shimoyama – Winter Milky Way | Spotify | Relaxing | Solo fingerstyle guitar | Slow | Min |
