# Supplementary material for: The relative contributions of subjective and musical factors in music for sleep
Source: PLoS One. 2025 Aug 21;20(8):e0330268. doi: 10.1371/journal.pone.0330268 (PMC12370070; doi:10.1371/journal.pone.0330268)
Supplement: Attachment — Submitted filename: PLOS-One_rebuttal.docx [file pone.0330268.s007.docx]

PLOS One response letter

Dear Dr. Kim,

Thank you for giving us the opportunity to submit a revised draft of our manuscript “The relative contributions of subjective and musical factors in music for sleep” for publication in the journal *PLOS One*. Our deepest appreciation to you and the reviewers for taking the time to read our manuscript and provide encouraging and constructive comments. Our responses are detailed below, and we submit a tracked copy of the manuscript highlighting our changes. We hope that we have properly and satisfactorily addressed all concerns and feedback.

We would also like to note that we have double checked the statistical analysis in the process of adding additional information to the supplementary material as requested by Review #2 and found that our results hadn’t factored in corrections for multiple comparisons; we have now corrected this, resulting in a change to not significant in two pairwise comparisons; one rating and one feature.

**Reviewer(s)' Comments to Author:**

**Reviewer #1:**

Well-structured, informative, and generally well-written comprehensive overview of the role of music in aiding sleep, particularly by exploring the subjective perceptions of the listener and the objective characteristics of the music that may contribute to its sleep-inducing qualities. These approaches combine subjective experiences with objective musical analysis, allowing us to understand how music can be used as a therapeutic tool to improve sleep. The presents organized sections, including an abstract, introduction, and detailed discussions on theoretical frameworks, methodologies, and results. Specifically:

i. Abstract:

The abstract concisely summarizes the study’s objectives, methods, and results, providing a quick understanding of the purpose and scope. It offers an overview of research on the role of music in promoting sleep and mentions key factors considered, such as the impact of musical characteristics on the listener’s sleep quality. A more explicit mention of the study’s specific perspectives could help further distinguish this work from previous similar research.

**Author response:** Thank you very much for this suggestion. We have added a couple of sentences to the abstract to emphasise this point (lines 34-38).

ii. Introduction:

The introduction provides context on the increasing prevalence of sleep disorders in modern society and highlights the potential of music as an intervention to improve sleep. It paves the way for the research by describing the historical use of music as a sleep aid and establishing the justification for scientifically studying this relationship. The research question is clearly presented, and the importance of understanding both subjective and objective factors involved in the effectiveness of music for sleep is emphasized. It could be interesting to elaborate on how existing research gaps are addressed by this approach, particularly regarding the integration of listeners’ subjective experiences and objective analysis of musical characteristics.

**Author response:** We have made small edits throughout the Introduction to emphasise the advantages of integrating subjective and objective analysis, and expanded the Current study section (lines 239-264) to better pinpoint our addressing of this gap (and address other comments by Reviewer #2).

iii. Study Design:

The study design is clearly presented, detailing the methods used to explore how listeners' subjective perceptions and the objective characteristics of music contribute to its ability to induce sleep. Including self-assessments from listeners and objective analyses of musical elements such as tempo, harmony, and rhythm is a strength, as it provides a holistic understanding of how music works as a sleep aid. More detailed explanations of the participants' demographic characteristics and parameters would enhance the study’s generalizability. Information on participants' culture, age ranges, gender distribution, and sleep habits allows readers to assess whether the results are applicable to a broader population and how, or if specific subgroups are more likely to benefit from music for sleep.

**Author response:** We have added details to the Methods and Materials section to explain the types of background information we gathered in this study (lines 345-353) and provided more details on our sample in the Results section (lines 471-478). We have also added descriptive statistics for all of our background measures to the Supplementary Material (see S2).

iv. Psychological Factors:

The discussion of psychological mechanisms influencing sleep, including wakefulness, emotion, and relaxation, is a strong point. By highlighting how music can regulate these factors, the text it connects the physiological and psychological aspects of sleep to music itself. It successfully explains how specific musical elements can influence the listener's emotional state, which in turn affects sleep onset and quality. This section could benefit from a deeper exploration of the neurobiological mechanisms in such an interaction, particularly how music interacts with brainwave patterns, waves, and autonomic nervous system activity during sleep. Further reflection on how music may affect different stages of sleep, such as REM and non-REM sleep, could also enrich the analysis.

**Author response:** Thank you for this suggestion. We appreciate that there is a dearth of research in the area of music and neuroscience, and indeed neural activity is a fundamental indicator of sleep behaviour. However, as the topic of this paper is not neuroscientific we feel that our literature review should be focused on aspects more specifically relevant to the theories and measures incorporated in the study.

v. Theoretical Frameworks:

The interdisciplinary approach draws on a variety of theoretical frameworks from music psychology, sleep research, and cognitive neuroscience, providing a flexible, broad, and comprehensive theoretical foundation. References to previous research on the effects of music on sleep are well integrated, linking this study to the existing literature. The work can benefit from a more explicit discussion on how this work builds upon or modifies previous theories. How does the study's focus on listeners' subjective perceptions contribute to or challenge established models of music’s impact on sleep, such as the "relaxation response theory" or the "entrainment theory"?

**Author response:** We have elaborated in the Discussion to discuss the contributions of this research to theories related to the use of music for sleep, and concepts related to music and emotions and the use of music for the purpose of wellbeing.

vi. Acronyms:

Some readers may not be familiar with terms like "MSSD" (Music Streaming Session Dataset), which could make the reading less accessible. The text could benefit from a very brief explanation or development when these terms are introduced for the first time (or even a later reminder - for example - for very specific citations/references). A dataset generated by users on musical preferences and listening habits related to sleep. In addition to any post footnotes or glossary appendixing, occasionally recalling/clarifying acronyms is a simple yet effective way to ensure accessibility for a broader, audience.

**Author response:** Thank you for pointing this out, as we only refer to the MSSD once we have removed this acronym and added details to our description of the source for clarity (lines 184-185). We have checked the document for any other use of acronyms and made similar amendments.

vii. Sentence Length and Structure:

Some sections contain long, complex sentences that may be difficult to follow for certain readers, particularly in the theory and result sections. Breaking these sentences into shorter, more digestible chunks would significantly improve the manuscript’s readability. When discussing specific theories or findings, it may be helpful to present each idea or concept separately, concisely, and in short sentences rather than grouping multiple ideas into one dense statement.

**Author response:** We have proofread the manuscript and made edits to improve the sentence structure and general flow of the document.

viii. Flow and Transitions:

Transitions between different sections, such as those between psychological theories and specific findings, present a challenge in the straightforward narration of the information we construct. A special revision is needed to maintain the logical flow of ideas and focus of attention, allowing readers to follow the narrative more easily. For example, after analyzing the psychological mechanisms involved in sleep, the results could more explicitly link these theoretical concepts by drawing on both ideas and expanding observed findings to strengthen the ties between theory and practice while creating a clearer narrative for the reader.

**Author response:** We have revised the article to improve the flow of language for the reader.

ix. Methodological Clarity:

There is still room to improve the methodology by providing more details, as it would make it more transparent to allow readers to assess study’s validity and applicability. Any additional information on participant recruitment is always welcome, and possible tendencies or unavoidable filters, as it would help readers understand the representativeness of the sample, and promote the sharing of these results and analyses ad hoc. The music selection process could be more detailed: was a specific music genre chosen, and how/why? How was the selection based on listener preferences?

**Author response:** We have added additional details to describe our sample demographics (lines 471-478), and used our Discussion to elaborate on our design limitations related to our participants and recruitment (lines 889-903). A detailed description of the music selection process is provided in the supplementary material, however we have also added details to the Stimuli part of the Methods and Materials section (line 273). We deliberately did not choose any specific genre with the ideal of gathering a mixed selection of music that broadly defines each category of music.

x. Musical Diversity and Impact:

An important consideration that could be addressed in the text is whether musical diversity, in terms of genre or cultural context and familiarity, was taken into account in the study’s design. The text could examine whether different genres (e.g., classical, ambient, jazz) or music from different cultures (listeners’ or others’) may have different effects on sleep. For example, how might an audience’s cultural context influence their perception of music from different origins and its effectiveness in inducing sleep? How can we measure or report such impressions? This seems an important emerging question, particularly in a globally interconnected world where people have access to a vast variety of music, a world that was previously extremely vast.

**Author response:** This is a very important limitation in our study and we greatly appreciate this point. As regards previous comments, we have tried to include more information on our sample in our reporting and in the Discussion section we have added comments to our sample limitations (lines 899-903). Specifically, we have mentioned the cultural significance of music for sleep that is evident in the prevalence of lullabies, and the open question of how this diverges into adulthood. A full investigation of individual differences was outside of the scope of this study, indeed our sample was notably skewed in several demographics. Ultimately, a proper evaluation of cross-cultural differences and other demographic variables would require a study with a different design, or much larger sample, with systematic consideration of stimuli, inclusion criteria and recruitment strategy (such as translation of the survey). This is something that we hope to pursue in future.

xi. Highlighting Novelty and Contributions:

The text summarizes existing literature on music and sleep but could explicitly highlight new/relevant/similar aspects based on previous research directions. Integrating listeners’ subjective perceptions alongside objective musical analysis can be a unique contribution to the field, which could be further emphasized in both the abstract and the introduction, as mentioned in the specific comments for such points, highlighting how this study fills a gap in current research and advances our understanding of how listener customization and preferences influence sleep outcomes.

**Author response:** Thank you for this reflection. We have made edits to emphasise this point throughout, and make a more explicit statement in the Implications section of our Discussion about the novelty of our approach and its contribution to the topic (lines 922-943).

xii. Practical Applications:

A more in-depth discussion on the practical applications of the results would enhance the findings and clarify how the results could inform the development of personalized sleep music technologies, compositions, music applications, or therapeutic interventions. How could sleep music recommendations be personalized based on each listener’s preferences, emotional responses, or sleep habits? What practical measures can music therapists, app developers, or healthcare providers take to integrate these results into concrete applications?

**Author response:** Alongside highlighting novelty and contributions (above) we also discuss the practical and theoretical implications of our research in the Implications section of our Discussion (lines 922-943).

The work contributes to the understanding of human sleep patterns and ways by exploring the interplay of subjective listener perceptions and objective musical features during sleep. It adds to the growing body of research on the use of music for health and well-being, particularly in the realm of sleep. The study's comprehensive design and integration of both psychological and musical elements provide a strong foundation for understanding how music can be used as a tool to improve sleep quality. The potential impact can be improoved if methodological ambiguities, dense writing, and a lack of emphasis on practical applications are better adressed through a clear explanation on to better focus upon novelty and the expanded discussion of practical usage. Therefore, the text could be further strengthened enhancing clarity, accessibility, and relevance, particularly in applied contexts like therapeutic music composition, music apps for sleep, and consumer-focused sleep aids.

**Author response:** Thank you very much for your feedback. We hope that we have addressed your concerns, and appreciate any further comments.

**Reviewer #2:**

Overall the authors present a study with a clear purpose, well executed while being aware of possible limitations and choices made for reasons of feasibility. The paper is well written and is supported by strong methodology.

My main question is the inclusion of the scale Distraction-Freeing the mind and the use of distraction as negative. I understand the complexity of the word distraction in that it could cover both negative distraction and positive distraction. For example, a track could distract from concentration and have a negative impact, as was meant with the inclusion of this scale. However, how about the case when individuals would use music to mask external sounds? Then distraction would be necessary and might have a positive effect on sleep. It would not be considered as Freeing the mind. Perhaps a scale from ‘Over-stimulation to Under-stimulation' of music would be more appropriate, as music could distract positively in cases of appropriate stimulation but negatively in cases of over-stimulation.

**Author response:** We indeed use distraction in this context as negative, looking at it from the perspective of the music being a distraction (or attention grabbing). This use relates to our focus on characterising the subjective properties of the music as experienced by the participant rather than the use of the music (see also response to your next comment). We have added a point in the Discussion that acknowledges that the questionnaire generally and poles used in the items require further validation (lines 904-911).

Furthermore, three reasons for using sleep are mentioned (arousal, valence and distraction). However, there are other possible reasons why individuals turn to music for sleep (such as masking) as proposed by Dickson & Schubert 2020 and Trahan et al. 2018. It would be good to see these mentioned with an explanation of why you are not taking these into consideration for this study.

**Author response:** Thank you for this point, this is indeed something we had not explicitly acknowledged. In our study we were interested in characterising the subjective properties or affordances of music, which we would distinguish from characterising the uses of music for sleep. There is of course a lot of overlap; the reasons for using music suggest what affordances music may have, and indeed Dickson & Schubert (2020) and Trahan et al. (2018) both refer to mood and distraction in their studies, for example. But we think there’s an important distinction to be made; masking is a reason why individuals might use music to help them sleep, but other sources could also meet that goal, such as listening to podcasts. This leads to a question of why someone might use music specifically; what affordances does music have that is different from other types of stimuli, why do individuals seem to have strong preferences for different types, and so one. We have added a new section on Mechanisms to the Discussion (lines 832-878) to consider this issue.

Finally, I question the role self-awareness for sleep music. I would think that needing to music to distract oneself would mean that they don’t want to have ruminating thoughts and would instead prefer to have an external stimulus to bring them outside of themselves. On L138, you mention that ‘dissociation’ is required for sleep. Isn’t dissociation the absence, or break of self-awareness?

**Author response:** This is a good point. We did not delve into this concept too much other than recognise that it is another concept referred to by Schäfer et al. (2013) that is relevant to music listening generally. We have reconsidered the phrasing of this section.

**Methods:**

I suggest having each analysis in a different paragraph for the ‘Analysis’ section of the methods and stating clearly the question investigated for each paragraph. As it stands, it’s a bit of a mess of normality statements and analyses which makes it hard to get a clear overview of how many analyses were done.

**Author response:** Thank you for this suggestion. We have amended the Analysis section in order to make it clearer.

I would like to know more about how you are certain that the included Sleep tracks were not commercial tracks. Spotify does have a certain number of commercial tracks or big sleep-music creators so I would like this to be described more.

**Author response:** We have clarified our stimuli reporting in the Methods and Materials section (lines 273-343); we did not check for this in our Spotify selection but the point of the additional sleep tracks was to use selections that also had some basis of use in previous studies. So, these were not only tracks that were commercially marketed for sleep and/or deep relaxation but also had some validation in the form of previous studies.

**Results:**

The results text-section about Figure 1 and Figure 1 is confusing to read as it assumes the reader remembers the opposite ends of each scale as listed in Table 1. As the authors state for example that ‘sleeping music was more calming than energising music’ but ‘Calming’ doesn’t appear in Figure 1. It would be nice if either the text only uses the words that appear in Figure 1, such as ‘less activating’ or to also include the opposite word in Figure 1. Similarly, adding two extra axes of Positive-Negative Valence and High-Low Arousal around the figure could visually illustrate what is said line 312-314. Maybe in the top right to avoid confusion with the axes of the graphs.

**Author response:** Thank you for pointing this out, we have edited the text as you have suggested.

Line 330 – consider also stating in which ways relaxing music was significantly different from energising music. Same at l. 336 for the musical features different between Relaxing and Energising music.

**Author response:** Thank you for the suggestion. We have added a sentence for each case in the Results (lines 499-51, 507-508).

Order of variables in Table 4 – For the combined model, it would be easier to read if they were in a logical order or if it was first the Ratings then the Features. Engaging also seems to be in the wrong place if the order is by significance level? I would also like to see the actual p and statistics to the supplementary material for the results of figures 1 and 2.

**Author response:** We have rearranged the table for Model C grouping the ratings and features separately and made sure they are ordered by significance and standardised coefficient values - this seems to have been a typo as it was done for Models A and B, thanks! We’ve also added the full statistical results to the supplementary material for all of the analyses (see S3).

Why was the linear analysis done while combining all categories together? Would it have not been interesting to do one for each category? Maybe there could be some argumentation about this choice.

**Author response:** The intention was to assess the music on a plane from not sleep inducing to sleep inducing, restrict the number of analyses conducted and avoid the potentially limiting group-based comparisons.

**Discussion:**

In the discussion of the role of familiarity, perhaps there may be a difference in the role of familiar music on the experience of music as sleep inducing and their experience of the music as relaxing and how it would actually make listeners feel?

**Author response:** There may indeed be interacting effects of familiarity and other subjective evaluations. We have added a sentence to our Discussion on Familiarity (lines 303-305), which was so far focused on more direct influences of familiarity. In our Mechanisms and Limitations sections of the Discussion we also mention how our results generally are based on perceptions in a listening study and it remains to be assessed how such appraisals compare to actual experiences at night-time (lines 872-878).

**Limitations:**

While the authors addressed certain limitations, I have a few points that could be added:

Firstly, the word Sedative is used in one of the rating scales. This is not a common word so non-native speakers (and perhaps native speakers!) might not have known its meaning.

**Author response:** We have added a general comment about the limitations of our choice of ratings to our Limitations section (lines 904-911).

Another limitation is the variance in the number of ratings each track received.

**Author response:** We have added a comment about this in our discussion (lines 912-915).

Finally, I have concerns about the exact terminology when referring to ‘sleep-inducing’ tracks in the last section of the results (around l.431 and in S2). By referring to the most sleep-inducing track, it seems as if participants actually fell asleep to these tracks –which is not the case. A suggestion for reformulation could be the tracks with the most sleep-inducing potential. Additionally, a disclaimer in the text around the last section in the discussion could point out this distinction and continue with the current terminology. In the discussion you say ‘the experience of music as sleep inducing’ which is perfect.

**Author response:** Thank you for picking up on this, we have been through the document and checked for consistency on this, making sure we’ve been clear on how ‘sleep-inducing’ is referred to.

**Small notes:**

L 54 in the referred paper, there were 985 playlists with 225,626 tracks

L. 68 ‘assessing’ instead of ‘to assess’

L. 78 Reverse the sentence starting with ‘We hypothesise that ...’ To make a less clunky sentence.

l. 159 is there a word missing? ‘Given the overlap with HOW? Music functions generally...’

L. 190 Who is the ‘they? Musicians? I see now that you meant the demographic information. Maybe this could be a bit clearer.

L. 198 to 200- I would suggest moving this to the next paragraph? I found it a bit confusing already being given information about the self-composed. I also wonder whether there was enough discussion/conclusion around this double purpose to warrant mentioning it here. Alternatively, a new paragraph could be added to the discussion about this double purpose.

L. 282 ‘Participants whose’.

Were the participants rewarded for their participation?

Line 325-326 is confusing.

First paragraph of discussion, some sentences are constructed in a complicated manner. I suggest re-reading and rephrasing some of them.

**Author response:** Thank you for pointing these out, we have been through and made the corrections. We have also added the clarification that participants were not rewarded for their participation (line 407).
