# Supplementary material for: The relative contributions of subjective and musical factors in music for sleep
Source: PLoS One. 2025 Aug 21;20(8):e0330268. doi: 10.1371/journal.pone.0330268 (PMC12370070; doi:10.1371/journal.pone.0330268)
Supplement: Attachment — Submitted filename: Reviewer comments.pdf [file pone.0330268.s005.pdf]

Overall the authors present a study with a clear purpose, well executed while being aware of possible limitations and choices made for reasons of feasibility. The paper is well written and is supported by strong methodology.

My main question is the inclusion of the scale Distraction-Freeing the mind and the use of *distraction* as negative. I understand the complexity of the word *distraction* in that it could cover both negative distraction and positive distraction. For example, a track could distract from concentration and have a negative impact, as was meant with the inclusion of this scale. However, how about the case when individuals would use music to mask external sounds? Then distraction would be necessary and might have a positive effect on sleep. It would not be considered as *Freeing the mind*. Perhaps a scale from 'Over-stimulation to Under-stimulation' of music would be more appropriate, as music could distract positively in cases of appropriate stimulation but negatively in cases of over-stimulation.

Furthermore, three reasons for using sleep are mentioned (arousal, valence and distraction). However, there are other possible reasons why individuals turn to music for sleep (such as masking) as proposed by Dickson & Schubert 2020 and Trahan et al. 2018. It would be good to see these mentioned with an explanation of why you are not taking these into consideration for this study.

Finally, I question the role self-awareness for sleep music. I would think that needing to music to distract oneself would mean that they don't want to have ruminating thoughts and would instead prefer to have an external stimulus to bring them outside of themselves. On L138, you mention that 'dissociation' is required for sleep. Isn't dissociation the absence, or break of self-awareness?

I would like to hear the authors' thoughts on these points.

Methods:

I suggest having each analysis in a different paragraph for the 'Analysis' section of the methods and stating clearly the question investigated for each paragraph. As it stands, it's a bit of a mess of normality statements and analyses which makes it hard to get a clear overview of how many analyses were done.

I would like to know more about how you are certain that the included Sleep tracks were not commercial tracks. Spotify does have a certain number of commercial tracks or big sleep-music creators so I would like this to be described more.

Results:

The results text-section about Figure 1 and Figure 1 is confusing to read as it assumes the reader remembers the opposite ends of each scale as listed in Table 1. As the

authors state for example that ‘sleeping music was more calming than energising music’ but ‘Calming’ doesn’t appear in Figure 1. It would be nice if either the text only uses the words that appear in Figure 1, such as ‘less activating’ or to also include the opposite word in Figure 1. Similarly, adding two extra axes of Positive-Negative Valence and High-Low Arousal around the figure could visually illustrate what is said line 312-314. Maybe in the top right to avoid confusion with the axes of the graphs.

Line 330 – consider also stating in which ways relaxing music was significantly different from energising music. Same at l. 336 for the musical features different between Relaxing and Energising music.

Order of variables in Table 4 – For the combined model, it would be easier to read if they were in a logical order or if it was first the Ratings then the Features. Engaging also seems to be in the wrong place if the order is by significance level?

I would also like to see the actual p and statistics to the supplementary material for the results of figures 1 and 2.

Why was the linear analysis done while combining all categories together? Would it have not been interesting to do one for each category? Maybe there could be some argumentation about this choice.

Discussion:

In the discussion of the role of familiarity, perhaps there may be a difference in the role of familiar music on the experience of music as sleep inducing and their experience of the music as relaxing and how it would actually make listeners feel?

Limitations:

While the authors addressed certain limitations, I have a few points that could be added:

Firstly, the word Sedative is used in one of the rating scales. This is not a common word so non-native speakers (and perhaps native speakers!) might not have known its meaning.

Another limitation is the variance in the number of ratings each track received.

Finally, I have concerns about the exact terminology when referring to ‘sleep-inducing’ tracks in the last section of the results (around l.431 and in S2). By referring to the most sleep-inducing track, it seems as if participants actually fell asleep to these tracks – which is not the case. A suggestion for reformulation could be the tracks with the most sleep-inducing potential. Additionally, a disclaimer in the text around the last section in the discussion could point out this distinction and continue with the current

terminology. In the discussion you say ‘the experience of music as sleep inducing’ which is perfect.

Small notes:

L 54 in the referred paper, there were 985 playlists with 225,626 tracks

L. 68 *‘assessing’* instead of *‘to assess’*

L. 78 Reverse the sentence starting with ‘We hypothesise that ...’ To make a less clunky sentence.

l. 159 is there a word missing? ‘Given the overlap with HOW? Music functions generally...’

L. 190 Who is the ‘they? Musicians? I see now that you meant the demographic information. Maybe this could be a bit clearer.

L. 198 to 200- I would suggest moving this to the next paragraph? I found it a bit confusing already being given information about the self-composed. I also wonder whether there was enough discussion/conclusion around this double purpose to warrant mentioning it here. Alternatively, a new paragraph could be added to the discussion about this double purpose.

L. 282 ‘Participants whose’.

Were the participants rewarded for their participation?

Line 325-326 is confusing.

First paragraph of discussion, some sentences are constructed in a complicated manner. I suggest re-reading and rephrasing some of them.
